# ASNet: Auto-Augmented Siamese Neural Network for Action Recognition

**DOI:** 10.3390/s21144720

**Published:** 2021-07-10

**Authors:** Yujia Zhang, Lai-Man Po, Jingjing Xiong, Yasar Abbas Ur REHMAN, Kwok-Wai Cheung

**Affiliations:** 1 Department of Electrical Engineering, City University of Hong Kong, Hong Kong, China; yzhang2383-c@my.cityu.edu.hk (Y.Z.); jingxiong9-c@my.cityu.edu.hk (J.X.); 2 TCL Corporate Research Co. Limited, Hong Kong, China; yasar.abbas@my.cityu.edu.hk; 3 School of Communication, The Hang Seng University of Hong Kong, Hong Kong, China; keithcheung@hsu.edu.hk

**Keywords:** action recognition, 3D-CNN, deep reinforcement learning, data augmentation

## Abstract

Human action recognition methods in videos based on deep convolutional neural networks usually use random cropping or its variants for data augmentation. However, this traditional data augmentation approach may generate many non-informative samples (video patches covering only a small part of the foreground or only the background) that are not related to a specific action. These samples can be regarded as noisy samples with incorrect labels, which reduces the overall action recognition performance. In this paper, we attempt to mitigate the impact of noisy samples by proposing an Auto-augmented Siamese Neural Network (ASNet). In this framework, we propose backpropagating salient patches and randomly cropped samples in the same iteration to perform gradient compensation to alleviate the adverse gradient effects of non-informative samples. Salient patches refer to the samples containing critical information for human action recognition. The generation of salient patches is formulated as a Markov decision process, and a reinforcement learning agent called SPA (Salient Patch Agent) is introduced to extract patches in a weakly supervised manner without extra labels. Extensive experiments were conducted on two well-known datasets UCF-101 and HMDB-51 to verify the effectiveness of the proposed SPA and ASNet.

## 1. Introduction

Video-based human action recognition is one of the key tasks in video understanding. It provides a wide range of applications [1,2,3,4,5] in intelligent surveillance, health care, human–computer interaction, robot learning, etc. Due to the availability of large-scale video datasets and the advances in deep learning technologies, such as deep convolutional neural networks (CNN) and LSTM [6,7,8,9], video-based action recognition has made significant progress in the last decade. In CNN-based action recognition algorithms, data augmentation is usually used to increase the diversity of samples. Random cropping is the most common data augmentation method to improve the generalization and robustness of the trained model [6,10,11,12]. However, it is found that the data augmentation methods based on random cropping often generate non-informative samples (video patches covering only a small part of the foreground or only the background). Basically, these samples can be considered as noisy samples with incorrect labels. These samples may confuse the supervised neural network training process, thereby reducing the performance of the action recognition accuracy.

As the videos in the commonly used action recognition datasets [6,13,14,15,16] are based on an aspect ratio between 1.3 and 1.8, isotropic resizing of input videos is usually required in the implementation of neural network training and inference [6,8,10,17,18,19,20,21,22].

**Figure 1:**
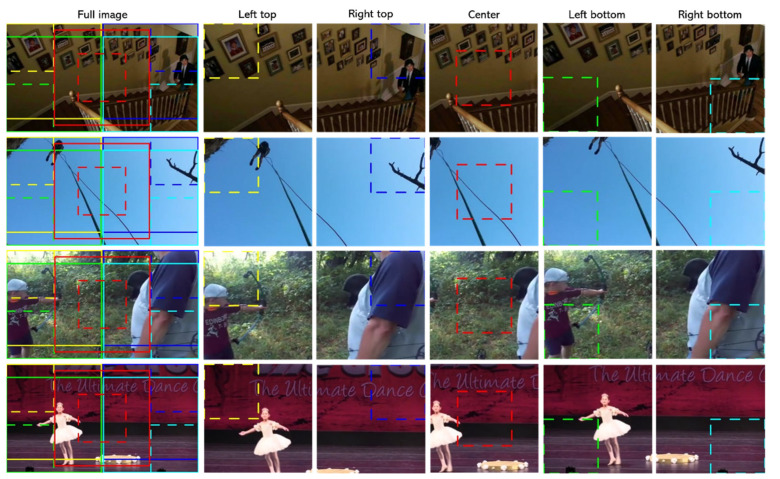
A schematic diagram for multi-ratio corner cropping on inputs. Multi-ratio corner cropping will randomly crop the four corners and the center with the size ratio 0.5∼1. Five different colors represent five different locations (Yellow: left top corner; Green: left bottom corner: Red: center; Navy Blue: right top corner; Light Blue: right bottom corner). The solid line is the original cropping size with a ratio 1.0. The dotted box represents a ratio 0.5 of the cropping size. Actions from the top to the bottom row are climbing stairs, abseiling, archery, and dancing ballet, respectively.

The neural network is fed with video patches, which are the randomly cropped samples from the input video frames. About 30–80% of the frame area may be lost in the cropping process. In the worst case, the cropped samples may have nothing to do with human action. For example, Figure 1 shows several multi-ratio corner cropping results using the four corners and the center of the video frames for randomly patch cropping with size ratios of 1 and 0.5. It is not difficult to observe that the process may generate many non-informative samples which are the patches covering only a small part of the foreground or only the background of the input video. If these samples are associated with the action labels of the input video, they would become noisy samples for neural network training because the context of these samples is not closely related to the corresponding action labels. In addition, it was verified in [23,24,25,26] that such noise samples may reduce the training performance of neural networks due to the introduction of wrong gradients direction.

Correspondingly, neural network inference also encounters a similar problem as center-cropping is adopted in inference [6,11]. If center-cropped input video preprocessing cannot cover the action context, it will be difficult for the neural network to recognize action based on the non-informative input patch. In order to improve the accuracy of inference, a common technique is to evenly crop three clips along the longer side of the input video [8,27]. However, this still cannot avoid the input of non-informative video patches, and the computational requirements of the inference process will also increase by three times. 

**Figure 2:**
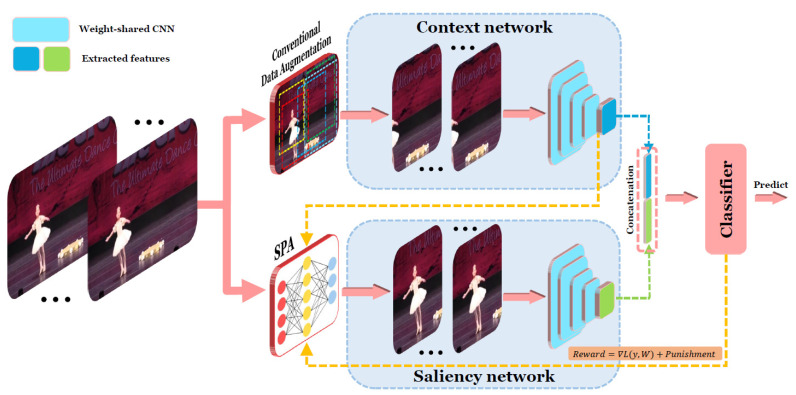
An overview of the proposed ASNet: ASNet is a two-stream CNN with shared weights. The top stream is the context stream. The bottom stream is the saliency stream. The context stream is fed with the clip cropped by conventional data augmentation while the saliency network is fed with the salient clip cropped by SPA.

Considering the above issues, we propose an Auto-augmented Siamese Neural Network (ASNet), which is trained using a reinforcement learning-based SPA (Salient Patch Agent) to reduce the negative impact of noisy samples generated during random cropping and to enhance salient information for action recognition. Figure 2 shows the network architecture of ASNet, which contains two shared-weight CNNs in context stream and saliency stream. The CNN in context stream receives input from data augmentation based on random cropping, and the CNN in saliency stream receives salient patches from SPA. A salient patch is defined as a spatial region in a video that contains critical information for action recognition.

In addition, we formulate the generation of salient patches as a Markov decision process. Using deep reinforcement learning to extract salient patches in a weakly supervised manner without extra labels provides an effective strategy to select the patches that can actively enhance the performance of ASNet for action recognition. In ASNet, the salient patches can compensate for the misleading gradient of non-informative samples in the training phase, thereby reducing the adverse effects of these samples. On the other hand, the CNN architecture aims to introduce attention in the final feature layer, which can enhance salient information in the inference stage. Extensive experiments were conducted to verify the effectiveness of the proposed SPA and ASNet on two well-known datasets UCF-101 and HMDB-51. In particular, the proposed method can achieve state-of-the-art performance on both datasets. To sum up, the main contributions of this work are four-fold:

- We addressed the issue of using random cropping methods for data augmentation in CNN-based video action recognition: generating noisy samples through random cropping will adversely affect the performance of the trained action recognition model.
- We proposed a Siamese neural network architecture that can reduce the negative impact of non-informative samples through gradient compensation and enhance critical information in the inference process.
- We proposed a new type of reinforcement learning agent, called SPA (Saliency Patch Agent), to generate salient patches. SPA can be weakly supervised to crop the critical information for action recognition from input video clips without additional labels.
- The proposed method has undergone end-to-end training and achieved state-of-the-art performance on UCF-101 and HMDB-51 datasets.

The rest of this paper is organized as follows. Section 2 provides a literature review of the most advanced methods in action recognition. Section 3 introduces our proposed method in detail. Section 4 discusses the experimental results. Section 5 provides conclusions.

## 2. Related Work

### 2.1. Deep Learning-Based Action Recognition

Before the widespread use of CNN-based techniques, traditional video action recognition methods are mainly based on handcrafted features [28,29,30,31,32]. Inspired by the impressive performance of deep learning in image classification and object recognition, CNN is widely used in action recognition and has already been dominant in this field. The CNN-based video action recognition framework can be summarized into five main families: two-stream architecture [33], 2D-CNN with temporal aggregation [10], 3D-CNN [17], convolutional RNN [34], and reinforcement architecture using attention mechanism and non-local structure [35].

More recent methods are the combinations of these architectures. Ji et al. [36] first designed 3D-CNN and applied it to the stack of frames, frame gradients and optical flow, thus verifying the effectiveness of CNN in video action recognition. Karpathy et al. [37] studied different fusion strategies of 2D-CNN semantic features to obtain spatiotemporal information with different input resolutions for action recognition. In order to make better use of temporal information, Simonyan and Zisserman [38] proposed a two-stream architecture composed of a spatial stream and a temporal stream. The system separately encodes spatial and temporal information, and then combines them in the last feature layer for classification. This method is considered to be a milestone for the CNN model to outperform traditional action recognition methods.

On the other hand, in order to encode long-term information of video, Donahue et al. [34] proposed a long-term recurrent convolutional network (LRCN) combining CNN and LSTM to learn perceptual representation and temporal dynamics at the same time. Tran et al. [17] extended 2D-CNN to 3D-CNN by introducing C3D neural network, which provides spatiotemporal feature extraction capabilities for the CNN models. In addition, to encode video-level information through 2D-CNN, Wang et al. [10] proposed a Temporal Segment Network (TSN) architecture and video-level prediction based on a two-stream method. In [39], Qiu et al. recycled off-the-shelf 2D networks for 3D-CNN, and studied different combinations of 2D-CNN and 3D-CNN to reduce the computational cost and memory requirements of 3D-CNN while improving the performance.

Based on 3D-CNN and two-stream architecture, Carreira and Zisserman [6] proposed two-stream inflated 3D ConvNet (I3D), which combined two-stream architecture with 3D-CNN and achieved the state-of-the-art performance. Tran et al. [18] mixed 2D-CNN and 3D-CNN, which resulted in a new spatiotemporal convolutional block R (2 + 1)D for action recognition. Zhou et al. [40] developed Temporal Relation Network (TRN) to enable 2D-CNN with inference ability to achieve better performance. In [35], Wang et al. were inspired by the classic non-local mean operation in computer vision and proposed a non-local structure that applies the attention mechanism in 3D-CNN. Xie et al. [41] proposed to replace 3D-CNN with low-cost 2D-CNN at the low-level layer of I3D, and suggested that temporal representation learning on high-level semantic features is useful. Feichtenhofer et al. [8] presented the SlowFast architecture to capture the semantic features of different video playback rates to improve performance. Lin et al. [42] proposed a temporal shift module (TSM) to shift the channel along the temporal dimension to integrate 2D-CNN based on temporal information. In [19], Feichtenhofer et al. proposed X3D—a group of efficient video networks to improve efficiency by expanding multiple axes in the features. Li et al. [22] proposed a channel-independent directional convolution to encode ordered temporal information at the clip level for action recognition.

Among these CNN-based action recognition methods, data augmentation with random-cropping derivatives is widely used. Although these data augmentation techniques increase the diversity of samples, they also generate non-informative samples, which is likely to degrade the overall recognition performance. In order to alleviate this shortcoming, we propose to use Siamese neural network architecture to mitigate the adverse effect of non-informative samples and SPA to detect salient patches as input to the network.

### 2.2. Data Augmentation

Volume and diversity of data are critical for deep learning models, but collecting labeled data is time-consuming and laborious. Therefore, data augmentation strategies were proposed to increase the diversity of existing data by applying various transformations, which turned out to be successful in training deep learning models.

Lecun et al. [43] applied several affine transformations, such as translation (horizontal and vertical), scaling, shearing for data augmentation for hand-written character recognition. Bengio et al. [44] applied more diverse transformations such as Gaussian blur, salt and pepper noise, Gaussian smoothing, motion blur, various occlusions. Krizhevsky et al. [45] applied random cropping, horizontal flipping, and color jittering (randomly changing color intensity) in AlexNet, which is a revolutionary work in image classification. Lemley et al. [46] proposed an end-to-end learnable augmentation process to decide the suitable augmentation method. DeVries and Taylor [47] proposed Cutout that randomly removes square regions of the input training images to improve the robustness of the model. Recently, Yun et al. [48] proposed CutMix which randomly cuts and mixes image patches among training samples where the image labels are also mixed proportionally. Based on CutMix, Uddin et al. [49] propose to use a saliency map to carefully pick salient training patches and mix this indicative patch with the target images. Gong et al. [50] used saliency maps to preserve salient informative regions during augmentation.

Random cropping-based data augmentation methods are comprehensively used in video action recognition. C3D [17], P3D [39], R (2 + 1)D [18], I3D [6] used random cropping to randomly crop fix-sized patches from isotropically resized videos; TSN [10], 3D ResNext [11], TSM [42], V4D [21], TEA [20] used multi-ratio corner cropping methods to randomly crop four corners and center with random size from isotropically resized videos. SlowFast [8], X3D [19] used multi-scale random cropping to random crop patches with random size from isotropically resized videos. In those data augmentation methods, it often generates non-informative samples which could be regarded as noisy labels which affect the overall performance. In this work, we propose ASNet to settle the problem. 

### 2.3. Saliency Detection for Action Recognition

The use of saliency detection to improve the performance of action recognition first appeared in [37] by Karpathy et al. To enhance action recognition performance, they proposed a two-stream network, in which one branch is used to resize the entire image, and the other branch is used for the center cropped image. In [51], Megrhi et al. made use of optical flow and clustering techniques to reduce the noise and camera motion, thereby generating saliency regions for large datasets. Xu et al. [52] applied a morphological gradient to RC-map for salient mask generation to improve dense trajectories, thereby enhancing the performance of action recognition. For action recognition, Tu et al. [53] proposed a human-related multi-stream CNN architecture with six CNN branches, in which the human detection algorithm is applied to salience detection of the saliency stream. Zhang et al. [54] proposed a Siamese Neural Network guided by motion patches based on optical flow to enhance motion information. Jiang et al. [4] proposed the use of Mask R-CNN detection to establish a saliency attention layer to eliminate CNN’s intra-frame redundancy. Tu et al. [55] proposed a combination of video object detection and motion saliency detection methods, which are based on pre-trained models from other datasets with extra labels to form a multi-stream neural network for action recognition. Weng et al. [56] utilized boundaries and optical flow to generate background-independent motion masks for action recognition.

On the other hand, there are two types of spatiotemporal-based saliency detection. One is to use handcrafted features, such as optical flow or iDT. The other one is to use pre-trained models for other tasks, such as human detection or object detection. Handcrafted features are susceptible to camera motion and environmental changes, while the performance of pre-trained detection models is easily affected by the original training datasets. In addition, saliency detection is also used in data augmentation in image recognition such as SaliencyMix [49] and KeepAugment [50]. Udding et al. also proposed to carefully select representative image patches and mix them with the target image with the help of saliency maps, so that the model can learn more appropriate feature representations. Gong et al. used saliency maps to measure the importance of each randomly cropped patch, and to avoid cropping saliency patches for region-level data augmentation.

In this paper, we propose an intelligent agent that uses policy learning to automatically learn where the salient regions are based on the loss output of the action recognition neural network under weak supervision without extra labels. Since the proposed saliency detection agent is trained on the main networks using the action recognition dataset, it can adapt to the distribution of the action recognition dataset and, thus, avoid the problem of using pre-trained models.

### 2.4. Deep Reinforcement Learning in Action Recognition

Deep reinforcement learning is a reinforcement learning framework based on deep learning, which was successfully applied to many computer vision applications [57,58,59,60]. Han et al. [61] first attempted to apply enhanced cropping agent learning to determine the video object segmentation scheme. Li et al. [59] proposed a weakly supervised aesthetic aware reinforcement learning framework to replace the sliding window mechanism to improve image cropping efficiency.

For action recognition, Dong et al. [62] proposed an attention-aware sampling agent based on deep reinforcement learning to select the most discriminative frame in the inference step to improve performance. Wu et al. [63] proposed a frame sampling agent based on multiagent reinforcement learning to drop non-informative frames of untrimmed video. Zheng et al. [64] used reinforcement learning agents to select effective segments for inference. Meng et al. [65] proposed to use reinforcement learning to select the optimal resolution for each frame in the video input for effective action recognition in long untrimmed videos.

Basically, traditional action recognition methods only use reinforcement learning for frame selection. However, in this paper, we treat salient patch clipping as a sequential decision-making process, and propose a new bounding box clipping strategy based on weakly-supervised reinforcement learning. While most patch selection methods based on reinforcement learning use sliding window methods, our proposed method directly determines the bounding box and only takes a few steps to complete the decision-making process. As far as we know, this is the first work to apply reinforcement learning agents in a weakly supervised manner to select salient patches in action recognition and to solve the problem of non-information samples in network training data augmentation.

## 3. ASNet Framework

The network architecture of the proposed ASNet framework is shown in Figure 2. The architecture consists of two CNN streams with shared weights. The top stream CNN is called context network, which receives input video patches generated by the traditional data augmentation method of video action recognition (i.e., random cropping for neural network training and center cropping for inference). We name it context network as it uses full information of input video through random cropping during network training with many iterations. Basically, context network plays the role of conventional single-stream CNN-based action recognition. The bottom stream CNN is called saliency network, which receives salient video patches extracted by SPA to increase the chance of capturing action-related information as input to the network. SPA is a reinforcement learning-based agent used to detect salient patches from the entire video scene. It is trained by the loss information from the action classifier output of the ASNet to ensure that the extracted regions are highly correlated with human actions.

### 3.1. Model Formulation

Let $X=\left\{X_{i}\right\},i\in \left[1,N\right]$ denotes the training dataset, where N is the total number of the videos in the training set and $X_{i}=\left\{x_{i1},x_{i2},\ldots ,x_{iG}\right\}$ is the $i_{th}$ video with G non-overlapping clips. $x_{ij}^{r}$ denotes the patches generated from the $j_{th}$ video clip by a conventional random cropping data augmentation method and $x_{ij}^{s}$ represents patches generated from the $j_{th}$ video clip in the $i_{th}$ video of the training set by the proposed SPA. $ℱ\left(x_{ij};W\right)$ is the function of ASNet with the parameters W, input $x_{ij}$, and output scores $s_{ij}=\left\{s_{ij}^{1},s_{ij}^{2},\ldots ,s_{ij}^{C}\right\}$, where C is the number of classes and $s_{ij}^{c}$ is the score of the $c_{th}$ class. In order to predict the likelihood, we use the normalization Softmax function S, which is computed as

(1) $$\begin{array}{c} {\bar{s}}_{ij}^{c}=\frac{e^{s_{ij}^{c}}}{\sum_{k=1}^{C}e^{s_{ij}^{k}}} \end{array}$$

where ${\bar{s}}_{ij}^{c}$ is the normalized score of the $c_{th}$ class. In addition, the loss function of the network with a regularized cross-entropy loss is given by

(2) $$ℒ\left(y,x,W\right)=-\overset{C}{\underset{k=1}{\sum }}y_{k}\log S_{k}\left(ℱ\left(x;W\right)\right)$$

where $y={\left(y_{1},\ldots ,y_{C}\right)}^{T}$ is the one-hot vector of the ground truth of the input x, and $S_{k}$ is equal to ${\bar{s}}_{ij}^{k}$. Therefore, ℱ(x;W) of ASNet can be expressed as

(3) $$ℱ\left(x;W\right)=C\left(G\left({ℱ}_{s}\left(x^{r},W^{s}\right),{ℱ}_{s}\left(x^{s},W^{s}\right)\right),W^{c}\right)$$

where ${ℱ}_{s}$ is the function of the weight-shared CNN and the well-known 3D ResNext [11] is used in our experiments as backbone CNNs. G is a feature combination function (e.g., sum, concatenation, multiply). C is a fully-connected neural network classifier. $W=\{W^{s},\;W^{c}\}$. $W^{s}$ represents the parameters of the shared weight in the context network and saliency network. $W^{c}$ represents the parameters of the classifier. To simplify the explanation, we denote $F_{s}^{p}$ as the feature maps activated by the information in the salient patch and denote $F_{s}^{o}$ as the feature maps activated by the information outside the salient patch. We assume that there is no information outside the salient patch in the saliency stream, that is, $F_{s}^{o}=0$ in the saliency stream. Substituting the symbols of these feature maps into Equation (3), we have

(4) $$ℱ\left(x;W\right)=C\left(G\left({ℱ}_{s}\left(\left\{F_{s}^{p};F_{s}^{o}\right\},W^{s}\right),{ℱ}_{s}\left(F_{s}^{p},W^{s}\right)\right),W^{c}\right).$$

For a single-stream neural network, it can be expressed as

(5) $$ℱ\left(x;W\right)=C\left({ℱ}_{s}\left(\left\{F_{s}^{p};F_{s}^{o}\right\},W^{s}\right),W^{c}\right).$$

Comparing Equation (4) to Equation (5), the proposed two-stream architecture of ASNet can obtain more information than a single-stream neural network. If SPA can provide action-related salient patches in the saliency stream, the performance of ASNet can be significantly improved as compared with single-stream architecture.

### 3.2. Salient Patch Agent

The key to achieving good performance of the proposed ASNet is to provide action-related salient video patches for the saliency network. This is realized through the deep reinforcement learning-based SPA, which can automatically extract salient patches from the input video clips, and then provide ASNet with critical information. However, it is not easy to extract salient patches from input video clips without additional labels and identify the most suitable region for action classification. To achieve this challenging patch extraction, a reinforcement learning agent relying on the deviation of the action classification loss as a reward is used. The cropping process of the salient patch is formulated as a Markov decision process, which uses a weakly supervised learning method to crop the patch without extra labels. The reward of SPA is calculated based on the loss of the fully connected neural network classifier C, which uses concatenated features [4,54] from the context network and saliency network as shown in Figure 2.

**Figure 3:**
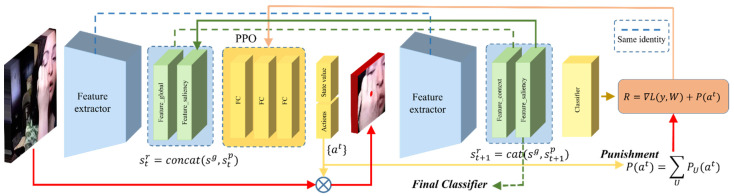
The framework of SPA: SPA starts with a feature extractor that shares the weights of the network of ASNet. The feature extractor is fed with a full scene frame clip and a salient clip made by SPA (the salient clip at the first step is initialized with a left corner with the size of 112 × 112) to generate two bags of features. Logits are obtained by feeding the concatenated two bags of features to the classifier of ASNet. Concatenate the two bags of features and the logits to generate a state $s_{t}^{r}$. Then the state is fed into the PPO agent for sampling actions, which guide to a new salient clip. Combine the full scene clip features with the new salient clip and feed the features to the classifier to obtain rewards. Form a new state, and the next cycle continues till the max step.

In this way, the agent can adjust the strategy to crop the patch, thereby reducing the loss of action classification. The architecture of the proposed SPA is shown in Figure 3. The system adopts the Actor–Critic model and directly regards the position and size of the bounding box of the salient patch as an action. With these settings, the process can be completed in just a few steps. The detailed description of SPA in terms of deep reinforcement learning terminology is as follows.

#### 3.2.1. State and Action Space

The state of SPA, $s_{t}^{r}$, consists of three components – $s^{g}$, $s_{t}^{p}$ and $s_{t}^{l}$. $s^{g}$ is the extracted feature of the full-scale input patch. $s_{t}^{p}$ is the extracted feature of a salient patch at step t. $s_{t}^{l}$ is the logits of the ASNet classifier based on the concatenation of $s^{g}$ and $s_{t}^{p}$. In the state $s_{t}^{r}$, $s^{g}$ provides the features of random cropping for SPA, which provides global information in multiple iterations and $s_{t}^{p}$ provides the features of action salient information in ASNet. Most cropping methods based on reinforcement learning use the sliding window approach. However, this approach needs moving and stretching the bounding box, which requires many steps to obtain accurate results. In the proposed SPA, we use a 3-action space $\left\{a_{m}^{t}|m=1,2,3\right\}$ to directly locate the bounding box. These three actions $\left(a_{1}^{t},a_{2}^{t},a_{3}^{t}\right)$ are the left corner location $\left(a_{1}^{t},a_{2}^{t}\right)$ and the length of the squared bounding box $a_{3}^{t}$, respectively. We set the actions range $a_{1}^{t}\in \left[0,w\right]$, $a_{2}^{t}\in \left[0,h\right]$, $a_{3}^{t}\in \left[0,min\left(w,h\right)\right]$, and (w,h) representing the width and height of the input frames, respectively.

#### 3.2.2. Reward

The reward represents the result value obtained through the agent’s interaction with ASNet. We calculate the reward based on the action classification output, and design the reward $r_{0}$ as

(6) $$r_{0}=\lambda_{1}sgn\left(ℒ\left(y,x,x_{t+1}^{p},\;W\right)-ℒ\left(y,x,x_{t}^{p},\;W\right)\right)+\lambda_{2}\underset{U=\left\{b,o\right\}}{\sum }P_{U}\left(a_{t}\right)$$

where $a_{t}$ is the action at step *t*, ℒ is the loss of the classifier with the concatenated features of x and the saliency patch $x^{p}$ extracted by SPA. $P_{U}$ is the punishment. $\lambda_{1}$ and $\lambda_{2}$ adjust the weights of loss deviation and punishment. We set two punishment rules, namely box size punishment and out of boundary punishment, to make SPA training converge faster and more stable. The punishments are defined as

(7) $$\begin{array}{ll} & P_{b}=\{\begin{array}{ll} & 0,\;\;\mathrm{if}\;a_{3}^{t}>=L\\ & \Omega ,\;\;\mathrm{otherwise} \end{array}\\ & P_{o}=\{\begin{array}{ll} & 0,\;\;\mathrm{if}\;a_{1}^{t}+a_{3}^{t}<=w\;\mathrm{or}\;a_{2}^{t}+a_{3}^{t}<=h\\ & \Omega ,\;\;\mathrm{otherwise} \end{array} \end{array}\;\;$$

where L is the threshold for box size punishment, and Ω is the punishment value. L is set as 56, and Ω is set as −5 through experimental tests.
**Algorithm1**. Training procedure of the SPA model$\mathrm{Input}:\;\mathrm{Original}\;\mathrm{input}\;\mathrm{frame}\;\mathrm{clips}\;x^{g}$Output: θ of SPA model$1:\;\mathrm{Initialize}\;x^{p}$, $\theta_{0}$, t=0$2:\;f_{global}=Feature\_extractor\left(x^{g}\right)$3: while k≤K**do**4:  while t≤T**do**$5:\;\;\;f_{saliency}=Feature\_extractor\left(x_{t}^{p}\right)$6:     Get *logits* through $f_{global}$, $f_{saliency}$
7:      $s_{t}^{r}=cat\left(f_{global},\;f_{saliency},logits\right)$
$8:\;\;\;\;\mathrm{Get}\;\left\{a_{m}^{t}\right\}$ from Policy $\pi \left(a_{t}|s_{t}^{r};\theta \right)$
$9:\;\;\;\;\mathrm{Crop}\;x^{g}$ by $\left\{a_{m}^{t}\right\}$ getting $x_{t+1}^{p}$
$10:\;\;\;r_{t}=reward\left(x^{g},x_{t}^{p},x_{t+1}^{p}\right)$$11:\;\;\;\mathrm{Estimate}\;\mathrm{advantage}\;{\hat{A}}_{t}$12:     t=t+1
13:   **end while**$14:\;\;\mathrm{while}\;j\leq N_{batch}$**do**$15:\;\;\;\;\mathrm{Calculate}\;{ℒ}_{\theta_{k}}^{CLIP}\left(\theta_{k}^{j}\right)$$16:\;\;\;\;\mathrm{Update}\;\theta_{k}^{j}$ with gradient $\nabla {ℒ}_{\theta_{k}}^{CLIP}\left(\theta_{k}^{j}\right)$
17:   **end while**18:   k=k+1
19: **end while**


**Algorithm 2. Training procedure of ASNet**
**Input**: Original input frame clips $x^{g}$**Output**: $\theta_{cnn}$ and $\theta_{spa}$1: Initialize $\theta_{cnn}$ and $\theta_{spa}$2: **while**
k≤K
**do**3:   Get $x^{c}$ through conventional cropping on $x^{g}$4:   Get $x^{s}$ through SPA on $x^{g}$5:   Take $x^{c}$ and $x^{s}$ as inputs; Fix $\theta_{spa}$; Train $\theta_{cnn}$6:   Fix $\theta_{cnn}$; Train $\theta_{spa}$ through Algorithm 17: **end while**

#### 3.2.3. Training of Salient Patch Agent

In SPA, we adopt the PPO algorithm with a clipped objective to train the patch selection policy. We designed the SPA model to share weights between the policy and value function. According to the setting of [58], the loss function is defined as

(8) $${ℒ}_{\theta }^{CLIP}\left(\theta \right)=\hat{E}\left[L_{t}^{CLIP}\left(\theta \right)-c_{1}L_{t}^{VF}\left(\theta \right)\right]$$

and

(9) $$\begin{array}{c} \begin{array}{c} \begin{array}{cc} L_{t}^{CLIP}\left(\theta \right)=\hat{E} & \left[min\left(r_{t}\left(\theta \right){\hat{A}}_{t},clip\left(r_{t}\left(\theta \right),1-\epsilon ,1+\epsilon \right){\hat{A}}_{t}\right)\right]\\ & L_{t}^{VF}\left(\theta \right)={\left(V_{\theta }\left(s_{t}\right)-V_{t}^{target}\right)}^{2} \end{array} \end{array} \end{array}$$

where the probability ratio is $r_{t}\left(\theta \right)=\frac{\pi_{\theta }(a_{t}|s_{t})}{\pi_{\theta_{old}}(a_{t}|s_{t})}$, θ is the network parameter of the SPA model, $\pi_{\theta }(a_{t}|s_{t})$ is the probability distribution of the policy under state $s_{t}$ and action $a_{t}$ at step t. We optimize the policy with minibatch AdamW. The estimated advantage function according to [66,67,68,69,70] is ${\hat{A}}_{t}=\sum_{i=0}^{T-t}\gamma^{i}r_{t+i}-V_{\theta }\left(s_{t}\right)$, where γ is the discount factor, $r_{t}$ is the SPA reward at step t, T is the number of steps of SPA. $V_{\theta }\left(s_{t}\right)$ is the value output with θ under state $s_{t}$. $V_{t}^{target}=\sum_{i=0}^{T-t}\gamma^{i}r_{t+i}$ represents the accumulated reward at step t.

## 4. Experiments

### 4.1. Experiment Settings and Implementation Details

#### 4.1.1. Datasets

The experiments were conducted on two well-known datasets-UCF-101 [13] and HMDB-51 [15] for video action recognition. UCF-101 is a dataset with three splits containing 13,320 videos from 101 action categories, avoiding non-motion frames. HMDB-51, which is more challenging than UCF-101, includes 7000 activity videos distributed across 51 action categories with natural disturbances with three splits.

#### 4.1.2. Training of CNN

The input frames were extracted at 25 fps and resized isotopically, with a minimum size of 256 pixels. We use RGB training settings in accordance with [11] and [7]. All the experiments were performed on a Pytorch platform with a GTX 2080Ti GPU. The backbone network is 3D ResNext-101 [9] (if not specified). The training process starts with a learning speed of 0.001. The batch size is 32. When the verification loss reaches a stable level, the learning speed is divided by 10. The weight decay was set to 1e-5 and used a stochastic gradient descent (SGD) optimization method with a momentum of 0.9.

The PPO model was trained with a learning rate of 0.0001, a weight decay of 1 × 10^−5^, and AdamW optimization for SPA. In addition, batch normalization [71] is applied to all convolutional layers. It should be noted that the weight initialization in SPA uses conventional initialization, which can make the training more stable. In order to generate input for the context stream of the proposed ASNet, we randomly selected 16 or 64 consecutive frames (16/64f-clip) from one video in the temporal dimension, and randomly sampled 224×224 crops with multi-scale corner cropping and random flipping in the spatial dimension according to [10]. Then the crops were resized into 112×112 as the inputs for the context stream.

For the saliency stream, we use the same clip as the context stream in the temporal dimension but spatially resize the inputs into 112×112 so that SPA can crop according to the entire scene of the clip. Then, SPA crops the salient patches from the original size clips according to the action context of the video and resize the salient patches to 112×112.

It took 2 days and 4 days to conduct the training with ResNext-101 with 64f samples on a 1080ti GPU on HMDB-51 and UCF-101, respectively. The response/inference time is about ~600 ms per 10 s video with ResNext-101 with 64 f samples on a 1080 ti GPU.

#### 4.1.3. Training of ASNet

The detailed training process of the SPA model and ASNet are elaborated in Algorithm 1 and Algorithm 2, respectively. K is the iterations for training SPA. N is the number of samples for minibatch Adam.$\;\theta_{cnn}$ and $\theta_{spa}$ are the parameters of the ASNet and SPA models, respectively.

#### 4.1.4. Inference Details

In the ASNet inference, we sampled non-overlapping 16f/64f-clips along the temporal dimension with center cropping in the spatial dimension for the context stream. For the saliency stream, the inputs were generated by SPA in the same way in training. We average scores of all non-overlapping inputs for the prediction.

### 4.2. Ablation Studies

#### 4.2.1. Comparison with Different Cropping Strategies

In the saliency stream of ASNet, we replaced SPA with various conventional data augmentation methods, and the action recognition results are shown in Table 1. Obviously, although the traditional data augmentation of random, corner, multiscale and center cropping methods can improve accuracy, their accuracy improvement is less than that of using SPA, especially on the HMDB-51 dataset. When saliency stream uses multi-scale cropped video patch input, the performance on HMDB-51 even decreases. In addition, we studied different fixed location cropping (fully resized, top left, top right, bottom left, bottom right) in the saliency stream of ASNet, and observed that the SPA strategy still outperforms them. The performance of fully resized video input is worse than that of SPA. A possible reason is that the fully resized video contains entire frame information, but the quality of the fully resized video is poor and contains a lot of useless background information, which can hurt the recognition performance.

#### 4.2.2. ASNet with Different Backbones

We compared the proposed ASNet with a single-stream network and a Siamese network with center cropping at the saliency stream (Siamese_center_) using different backbones on the split-1 of UCF-101 and HMDB-51 datasets. The results are shown in Table 2, which demonstrates that Siamese_center_ is better than the single-stream network using all the tested backbones. However, Siamese_center_ is still not compatible with ASNet using SPA. For UCF-101 and HMDB-51 datasets, the performance of ASNet with ResNext-101 (64 f) is improved by 1.2% and 3.6%, respectively, compared with the single-stream network, and the performance of Siamese_center_ is improved by 1.0% and 2.5%. In addition, we observed that as the network capabilities increase (i.e., deeper), the performance of ASNet will be better.

#### 4.2.3. ASNet with Different Feature Fusion Strategies

In this section, we compare five different fusion strategies referring to [54,72], such as Individual, Sum, Concatenation, Convolution and Multiply. The fusion layer is injected after the last convolutional layer since the features at that point are highly informative following [72]. In the Individual strategy, the features of the context stream and the saliency stream of ASNet were trained individually with the same fully connected layer (the classify layer) and the predictive scores of each stream were averaged for the final classification. Other strategies are the same as [54,72]. The comparison results can be seen in Table 3, where we report the accuracy on the first split of UCF-101 and HMDB-51 with 16 f clips. From the results in the table, we can conclude that although the feature fusion strategies benefit the performance, the Concatenation strategy performs better in our architecture. One possible reason for this may be that the final fully connected layer adaptively adjusts the weights of the features of the two streams of ASNet, which makes it a better performance. Thus, we adopt the Concatenation strategy in the proposed ASNet.

#### 4.2.4. Hyperparameters

In this section, we will show the experiments on SPA hyperparameters, that is, the number of actions and training steps required in SPA. Three different kinds of actions and four different numbers of training steps are conducted. The results are shown in Table 4 in which 2-action means that SPA has two actions $\left(a_{1},a_{2}\right)$ with spatial location $\left(a_{1},a_{2}\right)$ to determine the salient patch. The patch size is fixed and selected as the sample size 112. The 3-action means that SPA has three actions $\left(a_{1},a_{2},a_{3}\right)$ with location at $\left(a_{1},a_{2}\right)$ and the salient patch size $a_{3}\times a_{3}$. The 4-actions means that SPA has four actions $\left(a_{1},a_{2},a_{3},a_{4}\right)$ at location $\left(a_{1},a_{2}\right)$ with salient patch size of width $a_{3}$ and height $a_{4}$.

From Table 4, we can observe that the 3-action with 10 training steps perform best, although the 4-action with 10 training steps achieve the same performance as the 3-action in UCF-101. However, it is not comparable with the 3-action in HMDB-51. Basically, 3-action can maintain the aspect ratio of the input frame, and 4-action changes the input aspect ratio, which would affect the performance. Although 2-action can also maintain the ratio of the items, the size of the patch is fixed. However, if the item is larger than the predefined size, the prominent patch will lose external information. For different training steps, we can see that the 2-step training has the worst effect, and SPA tends to select a larger area in the frame under this step. The possible reason is that when there are too few training steps, SPA cannot obtain enough information to specify the critical information for action recognition, and thus cannot select more general regions. As the number of steps increases, we can see improvements in accuracy. However, when the training steps exceed 10, the performance stops improving further. We believe that the reason is that the 10 training steps have provided enough information for SPA to select an influential salient patch for ASNet.

Note that although the number of training steps is different, due to our strategy, when the SPA was trained, SPA strategy can quickly converge. So, we only need to perform two steps to test. Therefore, through this ablation study, we took three actions, 10 training steps and two test steps for SPA in other experiments.

### 4.3. Analysis of ASNet

In this section, the performance of ASNet and SPA will be analyzed in detail. First, we conducted two controlled experiments to explore the advantages of ASNet architecture. Then, the cropping performance of SPA in ASNet and the activation maps of ASNet are visualized. Finally, we analyze the action statistics of SPA on the HMDB-51 and UCF-101 datasets to prove its learning characteristics further.

#### 4.3.1. Exploration of ASNet Architecture

We used a single-stream CNN framework to compare with the proposed two-stream architecture of ASNet to demonstrate the advantages of co-training weights (backpropagated by randomly cropped patches and salient patches simultaneously), more salient inputs in ASNet. In order to show these enhancements, we designed two controlled experiments. In these experiments, the single-stream CNN and the two-stream ASNet with the same basic 3D ResNext [11] backbone were trained with the first split and 64 f-clips of the HMDB-51 dataset. 

For fair comparisons, the convolutional layers of these two networks and SVM are used as feature extractors and classifiers, respectively. The Top-1 action recognition accuracies of two networks on HMDB-51 dataset are shown in Table 5, in which $W_{s}$ is the weights of the CNN that trained by the single-stream neural network, $W_{a}$ is the weights of the shared-weight CNN that is trained by the two-stream ASNet. $X_{c}$ and $X_{s}$ denote the center cropping input and the inputs extracted by SPA, respectively. The final-layer features of ASNet are represented as $F_{a}$, which uses both the inputs of $X_{c}$ and $X_{s}$ for training. In addition, the final-layer features of single-stream networks that trained with center cropping input $X_{c}$ and SPA input $X_{s}$ are denoted as $F_{c}$ and $F_{s}$, respectively. 

First, the weights of the single-stream network $W_{s}$ and the weights of ASNet $W_{a}$ are compared. We use two new single-stream neural networks with the use of $W_{s}$ and $W_{a}$ (The shared weights of the ASNet of the two-stream network, thus it can be directly transferred to a single-stream backbone). As shown in Table 5, when center cropping (normal data preprocessing method [3]) is used in inference, the action recognition accuracy comparison is 75.0% ($W_{a}$) vs. 73.9% ($W_{s}$), where $W_{a}$ can achieve 1.1% improvement. While SPA cropping is used for inference, the action recognition accuracy comparison is 75.8 ($W_{a}$) vs. 74.5 ($W_{s}$), where $W_{a}$ can achieve 1.3% improvement. These denote that the weights ($W_{a}$) of ASNet outperform the weights ($W_{s}$) of the single-stream network and, thus, verifies that the two-stream-based ASNet with the use of co-training for shared weights can benefit the performance.

Secondly, the performances of using SPA cropping input $X_{s}$ and center cropping input $X_{c}$ are compared. We use weights of $W_{a}$ and $W_{s}$ to evaluate a new single-stream neural network with two different inputs $X_{s}$ and $X_{c}$. From Table 5 with the use of $W_{s}$, the action recognition accuracy comparison is 74.5 ($X_{s}$) vs. 73.9 ($X_{c}$), where $X_{s}$ can achieve 0.6% improvement. For the weights of $W_{a}$, the accuracy comparison is 75.8 ($X_{s}$) vs. 75.0 ($X_{c}$), where $X_{s}$ can achieve 0.8% improvement. These improvements demonstrate that the performance of using SPA cropping input is better than that of using traditional center cropping input.

**Figure 4:**
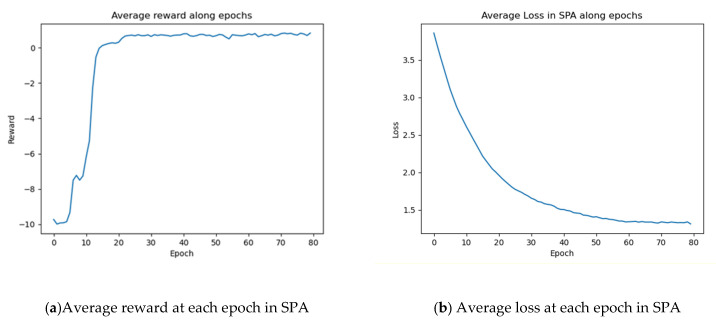
Average reward and loss at each epoch in SPA.

Furthermore, we plot the average reward and loss at each epoch in SPA to see the SPA training process in Figure 4. Average reward is the average reward of each taken action in SPA. Average loss means the average loss of each taken action in SPA. The experiment was conducted in the HMDB-51 dataset with 64 f training video clips. From these two figures, we can observe that the average reward of actions increases from 0 to 22 epochs and then jitters till the end and the average loss of SPA decreases gradually flatten out. This phenomenon shows that SPA can learn to get higher rewards in training and can be trained well with ASNet.

#### 4.3.2. Visualization of ASNet

**Figure 5:**
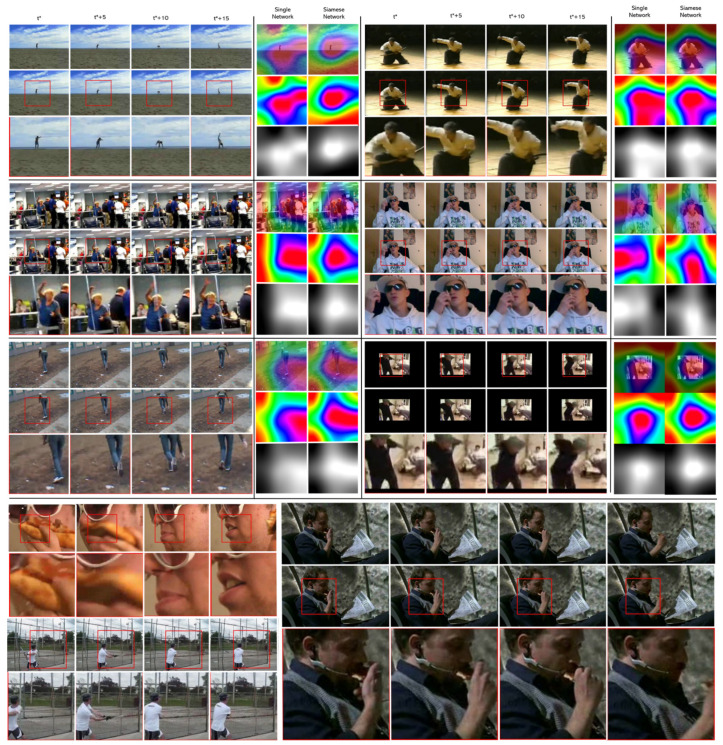
Visualization of ASNet: For each class, the pictures on the left hand are a sequence of frames of a video which are the inputs; the picture from top to bottom are original frames, original frames with cropping boxes of SPA, and the cropped images by SPA, respectively. The pictures on the right hand are Grad-CAM heatmaps of the inputs in a single-stream network and ASNet, respectively.

The cropping performance of SPA and Grad-CAM [73] of ASNet are shown in Figure 5. First, we observe that SPA tends to select most of the patches that contain the motion part of the input frames. Secondly, from the image in the upper left corner, it can be seen that SPA is selecting not only the human, but also the critical patch for action recognition from the picture in the complex background. Thirdly, the traditional data preprocessing method for inference is to isotropically resize input frames and then crop the center of the frames. However, when the long side of the image is much larger than the short side, the critical information will be lost. The examples shown in Figure 5 demonstrate that SPA is possible to avoid the loss of critical information for action recognition. In addition, by comparing Grad-CAM, we can find that ASNet’s Grad-CAM mapping is more action-specific than single-stream neural networks. This phenomenon verifies the effectiveness of ASNet. It should be noted that from Figure 5, some actions are not in the center of the bounding box. We believe that this phenomenon is reasonable because the conventional convolutional operation is not location-aware, i.e., the highlighted information of CNN is not related to the location on the inputs. 

### 4.4. Comparison with the State of the Art

In this section, we compare ASNet with the state-of-the-art action recognition methods using the three splits of UCF-101 and HMDB-51 based on 64 f-clips. The action recognition accuracies of these well-known methods are shown in Table 6. GFLOPs × Views represents the FLOPs per view in the 10-s video, which is the normal duration of the action recognition datasets. It is worth noting that the proposed model only uses the center crop in the spatial dimension, and continuous non-overlapping clips in the temporal dimension. Table 6 shows that the proposed ASNet model could reach state-of-the-art performance on both UCF-101 and HMDB-51 datasets. When using a single-stream network with 3D ResNext, only 95.1% and 73.4% can be achieved on UCF-101 and HMDB-51, respectively. These accuracies are 0.3% and 1.1% lower than I3D, 1.7% and 1.1% lower than R(2 + 1)D, and 1.4% and 2.5% lower than S3D. However, when ASNet is used with 3D ResNext, better accuracies can be obtained. ASNet’s performance is 1.4% higher than that of I3D, and it matches the performance of R(2 + 1)D and S3D on UCF-101. The performance is also better than I3D, R(2 + 1)D, and S3D by 1.9%, 1.9%, and 0.5% on HMDB-51 dataset, respectively. For HMDB-51, ASNet outperforms all these conventional methods with naïve 3D ResNext. We can observe that the improved performance on HMDB-51 is more prominent than UCF-101. One of the reasons is that HMDB-51 contains a larger aspect ratio video, and the main body of the action in HMDB-51 is different from UCF-101. The ratio of main bodies of actions locating at the center in UCF-101 is more than the ratio in HMDB-51. This phenomenon demonstrates the effectiveness of SPA to extract salient video patches for the ASNet to perform action recognition. In addition, as ASNet shares weights in the network and SPA is made of the three-layer perceptron, the number of additional parameters of ASNet is less than 1% (0.475 M) of its backbone 3D ResNext (48.34 M). Overall, the results in Table 6 show that the proposed ASNet can achieve state-of-the-art performance on UCF-101 and HMDB-51 with fewer total FLOPs.

## 5. Conclusions

In this paper, we addressed the issue of noisy samples generated in data augmentation of CNN-based video action recognition. Traditional random and center video patch cropping methods may generate many non-informative samples that only contain a small part of the foreground or even only covering the background area. These noisy samples may greatly degrade the neural network training quality as well as reduce the inference accuracy of the action recognition. To alleviate this issue, ASNet using Siamese CNN architecture and SPA (Saliency Patch Agent) based on reinforcement learning for video action recognition is proposed. The Siamese network architecture consists of a context network and a saliency network. The context network preserves features extracted from traditional random or center cropping video patch input while the saliency network increases the chance of extracting human action-related features from video patches provided by SPA. Weak supervision without extra labels is used to train SPA, and the deviation of ASNet’s action classification loss is used as a reward for reinforcement learning. Then, SPA can learn to crop the salient patches for improving the action recognition accuracy. Experiments were conducted to verify the effectiveness of the proposed ASNet framework using SPA and demonstrate that ASNet can achieve state-of-the-art action recognition performance.

## Figures and Tables

**Table 1:** Comparison with different cropping strategies in the saliency stream of ASNet. Top-1 accuracy using 16f clips of split-1 of UCF-101 and HMDB-51. The experiment’s backbone is 3D ResNext-101.

Cropping Strategy	UCF-101 (%)	HMDB-51 (%)
Baseline	91.7	66.7
Random-cropping	92.2	66.8
Corner-cropping	92.3	67.0
Multiscale-cropping	91.9	64.7
Center-cropping	92.5	67.6
SPA-cropping	**93.7**	**69.2**
Fully-resize	92.3	67.2
Left Top Corner	92.1	66.9
Right Top Corner	91.5	67.0
Center	92.5	67.6
Left Bottom Corner	92.0	67.1
Right Bottom Corner	92.2	67.1

**Table 2:** Comparison with different backbones. Top-1 accuracy using 16 f clips of split-1 of UCF-101 and HMDB-51.

Backbone	Single Stream		Siamese_center_		ASNet	
	UCF-101	HMDB-51	UCF-101	HMDB-51	UCF-101	HMDB-51
ResNet-18	84.5	57.3	85.0	57.5	86.7	57.5
ResNet-50	88.7	62.4	88.8	62.4	90.5	62.4
ResNet-101	88.6	63.6	88.9	63.8	90.6	64.7
DenseNet-121	87.5	61.1	88.1	61.3	90.1	61.7
ResNext-101	91.7	66.7	92.1	67.0	93.7	69.2
ResNext-101 (64 f)	95.2	74.1	95.4	75.2	96.4	77.7

**Table 3:** Comparison with different feature fusion methods. Top-1 accuracy using 16 f clips of split-1 of UCF-101 and HMDB-51. The experiment’s backbone is 3D ResNext-101.

Fusion Strategy	UCF-101 (%)	HMDB-51 (%)
Baseline (single branch)	91.7	66.7
Individual	92.8	68.1
Sum	92.7	67.8
Concatenation	**93.7**	**69.2**
Convolution	92.6	67.8
Multiply	92.1	66.8

**Table 4:** Performance evaluation of multiple actions and steps of SPA in ASNet. Top-1 accuracy using 16 f clips of split-1 of UCF-101 and HMDB-51.

Steps	UCF-101 (%)				HMDB-51 (%)			
	2	5	10	15	2	5	10	15
2-actions	92.4	92.5	92.8	93.0	67.5	67.8	67.8	67.9
3-actions	93.1	93.6	93.7	93.4	68.3	68.5	69.2	68.7
4-actions	92.9	93.2	93.7	93.5	67.9	68.2	68.5	68.5

**Table 5:** Exploration of each enhancement of ASNet. Top-1 accuracy using 64 f clips of split-1 of HMDB-51.

	HMDB-51 (%)		
	Single-Stream Neural Network		ASNet
	Center Crop $\left(X_{c}\right)$	SPA Crop $\left(X_{s}\right)$	Center and SPA Crop $(X_{c}$ and $X_{s})$
$W_{s}$	73.9 (baseline)	74.5	75.5
$W_{a}$	75.0	75.8	76.6

**Table 6:** Comparison with the state of the art. Top-1 accuracy of the mean accuracy across three splits of UCF-101 and HMDB-51.

Methods	Input Size	GFLOPs × Views	UCF-101	HMDB-51
C3D [17]	224 × 224	296.7 × 4	85.2	51.6
Res3D [74]	224 × 224	-	85.8	54.9
P3D [39]	224 × 224	-	88.6	-
3D-ResNext [11]	112 × 112	48.4 × 4	95.1	73.4
MRST-T [75]	224 × 224	99.6 × 4	96.5	75.4
StNet [76]	256 × 256	310.5 × 4	94.3	-
iDT-RCB [52]	-	-	94.8	-
STSAMANet [4]	128 × 128	-	95.9	-
STSVOS [55]	224 × 224	-	93.9	67.2
ATEN [56]	-	-	94.6	70.5
STS-ALSTM [77]	-	-	92.7	64.4
RSTAN [78]	-	-	94.6	70.5
TSN [10]	224 × 224	3.2 × 250	93.2	-
TSM [42]	224 × 224	65 × 30	95.9	73.5
STM [79]	224 × 224	66.5 × 30	96.2	72.2
TEINet [80]	224 × 224	66 × 30	96.7	72.1
DropPath [81]	224 × 224	254 × 2	96.5	-
I3D [6]	224 × 224	107.9 × 4	95.4	74.5
S3D [41]	224 × 224	66.4 × 30	96.8	75.9
R(2 + 1)D [18]	112 × 112	152.4 × 4	96.8	74.5
DSN [64]	112 × 112	158 × 4	96.8	75.5
ASNet	112 × 112	104.5 × 4	**96.8**	**76.4**

## Data Availability

UCF-101 [13]: https://www.crcv.ucf.edu/data/UCF101.php (accessed on 10 July 2021); HMDB-51 [17]: https://serre-lab.clps.brown.edu/resource/hmdb-a-large-human-motion-database/ (accessed on 10 July 2021).
